# XBP1 modulates endoplasmic reticulum and mitochondria crosstalk via regulating NLRP3 in renal ischemia/reperfusion injury

**DOI:** 10.1038/s41420-023-01360-x

**Published:** 2023-02-17

**Authors:** Haiqiang Ni, Zhiyu Ou, Yuchen Wang, Yanna Liu, Kailun Sun, Ji Zhang, Jiasi Zhang, Wenfeng Deng, Wenli Zeng, Renfei Xia, Jian Xu, Nianqiao Gong, Yun Miao

**Affiliations:** 1 Department of Transplantation, Nanfang Hospital, Southern Medical University, 510515 Guangzhou, China; 2 Department of Gastroenterology and Hepatology, Beijing Youan Hospital, Capital Medical University, 100069 Beijing, China; 3 Institute of Organ Transplantation, Tongji Hospital, Tongji Medical College, Huazhong University of Science and Technology, 430030 Wuhan, China; 4 Key Laboratory of Organ Transplantation of Ministry of Education, National Health Commission and Chinese Academy of Medical Sciences, 430030 Wuhan, China

**Keywords:** Cell death, Urogenital diseases

## Abstract

The functional status of mitochondria and the endoplasmic reticulum are central to renal ischemia/reperfusion injury (IRI). X-box binding protein 1 (XBP1) is an important transcription factor in endoplasmic reticulum stress. NLR family pyrin domain containing-3 (NLRP3) inflammatory bodies are closely related to renal IRI. In vivo and in vitro, we examined the molecular mechanisms and functions of XBP1-NLRP3 signaling in renal IRI, which influences ER-mitochondrial crosstalk. In this study, mice were subjected to 45 min of unilateral renal warm ischemia, the other kidney resected, and reperfusion was performed for 24 h in vivo. In vitro, murine renal tubular epithelial cells (TCMK-1) were exposed to hypoxia for 24 h and reoxygenation for 2 h. Tissue or cell damage was evaluated by measuring blood urea nitrogen and creatinine levels, histological staining, flow cytometry, terminal deoxynucleotidyl transferase-mediated nick-end labeling, diethylene glycol staining, and transmission electron microscopy (TEM). Western blotting, immunofluorescence staining, and ELISA were used to analyze protein expression. Whether XBP1 regulates the NLRP3 promoter was evaluated using a luciferase reporter assay. Kidney damage was reduced with decreasing blood urea nitrogen, creatinine, interleukin-1β, and interleukin-18 levels. XBP1 deficiency reduced tissue damage and cell apoptosis, protecting the mitochondria. Disruption of XBP1 was associated with reduced NLRP3 and cleaved caspase-1 levels and markedly improved survival. In vitro in TCMK-1 cells, XBP1 interference inhibited caspase-1-dependent mitochondrial damage and reduced the production of mitochondrial reactive oxygen species. The luciferase assay showed that spliced XBP1 isoforms enhanced the activity of the NLRP3 promoter. These findings reveal that XBP1 downregulation suppresses the expression of NLRP3, a potential regulator of endoplasmic reticulum mitochondrial crosstalk in nephritic injury and a potential therapeutic target in XBP1-mediated aseptic nephritis.

## Introduction

Acute kidney injury (AKI) is associated with severe morbidity and mortality and is prevalent globally. However, the mechanisms underlying renal dysfunction and tissue damage remain to be fully elucidated [1]. Ischemia/reperfusion, a common cause of AKI [2], involves inflammation, hemodynamic alterations, and epithelial and endothelial cell death [3, 4]. Accumulating evidence reveals that ischemia/reperfusion injury (IRI) is a multifactorial process in which the mitochondria and endoplasmic reticulum (ER) play central roles [5]. Recent studies have shown that injury to the ER or mitochondria could disrupt ER-mitochondrial crosstalk in the kidneys, leading to renal injury. Thus, ER-mitochondria crosstalk may be a potential therapeutic target in AKI [6].

A dysfunctional ER may contribute to the accumulation of unfolded or misfolded proteins during IRI, causing ER stress (ERS). Upon the detection of unfolded proteins, three transmembrane receptors in the ER, namely ATF6, IRE1α, and PERK, activate the unfolded protein response signaling pathway [7–9]. Low levels of the unfolded protein response protect the kidneys from renal IRI [10]. However, severe ERS induces the activation of cell death cascades [11, 12]. ERS leads to the opening of the mitochondrial permeability transition pore (mPTP), which then depletes the mitochondrial membrane potential (MMP) and triggers the release of mitochondrial pro-apoptotic signals [13], reduction in MMP, and release of cytochrome c (Cyt-c) and other apoptosis-related factors. In particular, AKI-induced mitochondrial reactive oxygen species (mROS) promote the activation of the NLRP3 inflammasome, leading to caspase 1-dependent production of gasdermin D (GSDMD)-mediated pyroptosis and the maturation of the pro-inflammatory cytokines interleukin (IL)-1β and IL-18 [14–16].

Recent studies have examined the regulatory functions of the mitochondria-associated ER membranes (MAMs). Aside from ER chaperones, other inflammatory factors such as sigma-1 receptor [17], IP (3) receptor [18], GRP78 [19], calnexin [20], IRE1α, and PERK can also modulate ER-mitochondrial crosstalk [21–23]. When the mitochondrial fission protein, fission 1 homolog (Fis1), binds to B cell receptor-associated protein 31 (BAP31) located in the MAMs and forms the Fis1-BAP31 complex, cell death signals are conveyed to the ER lumen, initiating the cell apoptosis pathway [24]. Under ERS, unspliced X-box-binding protein 1 (XBP1u) mRNA (a non-transcriptional form) is activated by the RNase activity of IRE1α, which excises 26 nucleotides, causing a frameshift and forming a transcriptionally spliced XBP1 (XBP1s) mRNA [25]. The transcription factor XBP1s then regulates a series of important target genes to determine cell fate [26]. XBP1 downregulation has been found to reduce renal IRI by inhibiting HRD1-mediated NRF2 ubiquitination [27]. Although XBP1 downregulation under severe stress is known to protect tissues and organs, how (or even whether) XBP1 participates in the ER-mediated regulation of mitochondria or MAMs in renal IRI remains unknown. To address this, we examined how the XBP1-NLRP3 axis modulates ER-mitochondrial crosstalk during renal IRI in vivo and in vitro.

## Results

### IRI triggers severe ERS and markedly increases XBP1s expression

**Fig. 1:**
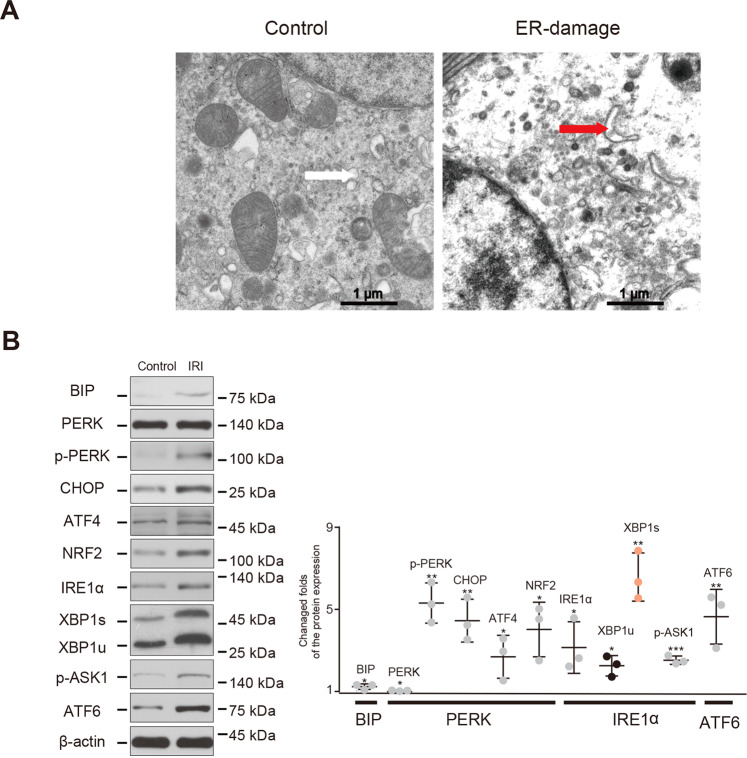
IRI triggers severe ERS and noticeable XBP1s expression. To construct the mouse IRI model, we used microvascular clips to block the left renal pedicle of the mouse for 45 min and cut off the right kidney after opening the bloodstream. **A** TEM analysis showed ER damage was induced by IRI. The white arrow indicates the normal ER, while the red arrow indicates swollen ER lumens. Scale bar = 1 μm; ×5000 magnification. **B** The expression levels of ER stress markers were detected using western blotting. The protein level of XBP1s was considerably higher than that of other molecules after IRI, as shown by the fold-changes. *P < 0.05, **P < 0.01, ***P < 0.001 vs. The normal control group (*n* = 3 samples/group). IRI ischemia/reperfusion injury, ER endoplasmic reticulum.

To detect ER morphology in IRI kidney sections, ultrastructural analysis was performed using TEM. Abnormal ER morphology, manifested as distinct vacuolation and swelling (characteristic of ERS), was observed (Fig. 1A). To further evaluate ERS, western blotting was used to analyze the expression of key ERS markers in the BIP and IRE1α pathway (IRE1α, XBP1u, XBP1s, and p-ASK1), PERK pathway (PERK, p-PERK, CHOP, ATF4, and NRF2), and the ATF6 pathway. The fold-changes in expression relative to the control were 1.24 ± 0.08 (*P* < 0.05) for BIP, 3.14 ± 0.73 (*P* < 0.05) for IRE1α, 2.25 ± 0.29 (*P* < 0.05) for XBP1u, 6.04 ± 1.36, (*P* < 0.05) for XBP1s, 2.53 ± 0.12 (*P* < 0.001) for p-ASK1, 1.04 ± 0.01 (*P* < 0.05) for PERK, 5.31 ± 0.57 (*P* < 0.01) for p-PERK, 4.29 ± 1.03 (*P* < 0.05) for CHOP, 2.69 ± 0.60 (*P* < 0.05) for ATF4, 3.89 ± 0.90 (*P* < 0.05) for NRF2, and 5.11 ± 1.00 (*P* < 0.05) for ATF6. Notably, the fold-change was significantly greater for XBP1s than for the other proteins, indicating that this activated transcription factor may play a key role in response to IR-induced kidney injury.

### IRI induces mitochondrial injury and NLRP3 inflammasome activation

**Fig. 2:**
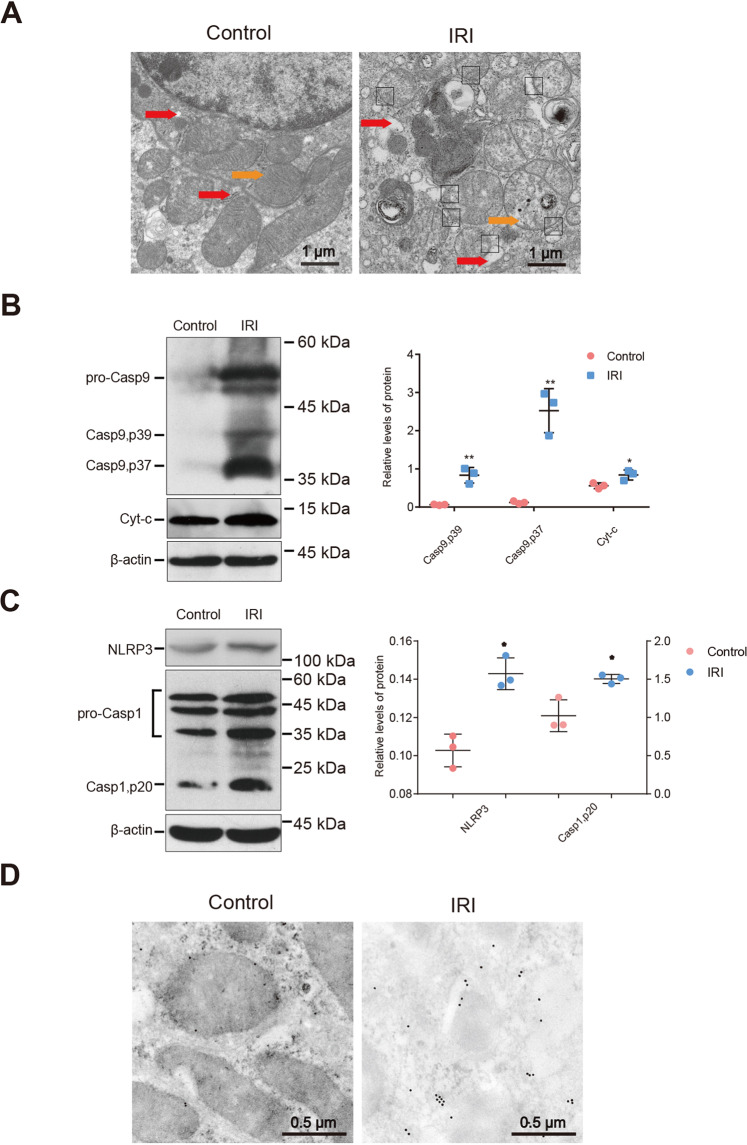
IRI induces mitochondrial injury and NLRP3 inflammasome activation. To construct the mouse IRI model, we used microvascular clips to block the left renal pedicle of the mouse for 45 min and then cut off the right kidney after opening the bloodstream. **A** ERS and mitochondrial damage developed simultaneously. TEM analysis showed that the ER maintained an appropriate distance or formed a direct connection with the mitochondria. The white arrow indicates normal ER, the black arrow indicates normal mitochondria, the red arrow indicates ER swelling, the orange arrow indicates damaged mitochondria, and the blank rectangle indicates MAMs. Scale bar = 1 μm; ×5000 magnification. **B** The protein expression of caspase-9 and total Cyt-c were increased in the IRI group. Western blot; *P < 0.05, **P < 0.01 vs. the normal control group (*n* = 3 samples/group). **C** The expressions of NLRP3 and caspase-1 were increased in the IRI group. Western blot; *P < 0.05 vs. the normal control group (*n* = 3 samples/group). **D** IEM analysis showed more NLRP3 translocated to the mitochondria or MAMs after renal IRI. The red arrow indicates the ER, the orange arrow indicates the mitochondria, the blank rectangle indicates the MAMs, and the small black dots indicate 10-nm colloidal gold particles. Scale bar = 0.5 μm; ×10,000 magnification. IRI ischemia/reperfusion injury, ER endoplasmic reticulum, MAMs mitochondria-associated endoplasmic reticulum membranes.

Mitochondrial damage and the NLRP3 inflammasome play important roles in ischemia-reperfusion injury [27–29]. TEM confirmed mitochondrial damage, characterized by mitochondrial swelling, cristae fracture, membrane damage, and abnormal ER in the vicinity of the MAMs (Fig. 2A). The relative expressions of the mitochondrial damage markers total Cyt-c and caspase-9 (Casp9) were significantly elevated relative to the control (Cyt-c: 0.56 ± 0.04 vs. 0.84 ± 0.08, *P* < 0.05; Casp9, p39: 0.06 ± 0.01 vs. 0.83 ± 0.12, *P* < 0.01; Casp9, p37: 0.12 ± 0.02 vs. 2.53 ± 0.33, *P* < 0.01; Fig. 2B). Western blot analysis revealed a significantly elevated expression of NLRP3 molecules and caspase-1 activators in the IRI group relative to the control (0.10 ± 0.01 vs. 0.14 ± 0.01 and 1.02 ± 0.12 vs. 1.50 ± 0.03, respectively*, P* < 0.01; Fig. 2C), indicating inflammasome activation. In addition, immunoelectron microscopy (IEM) revealed an enhanced NLRP3 colocalization with mitochondria under IRI, and that NLRP3 may even be able to translocate to the MAMs (Fig. 2D).

### The molecular mechanism of the crosstalk between ERS and mitochondrial dysfunction in response to IRI is mediated by the XBP1-NLRP3 axis

**Fig. 3:**
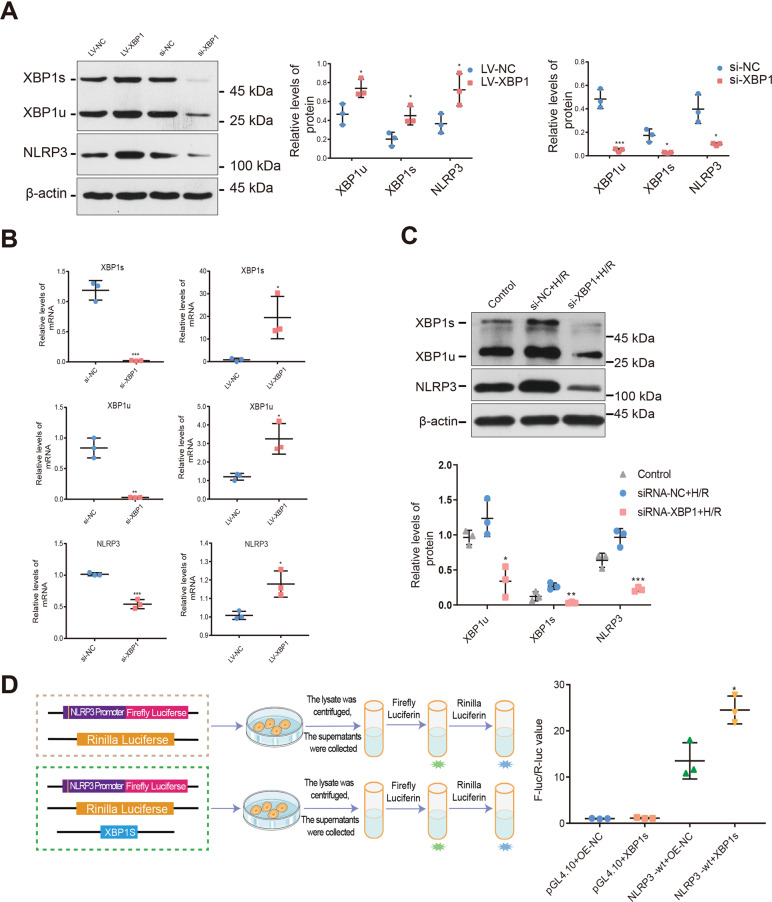
The XBP1-NLRP3 axis forms a molecular mechanism involved in the crosstalk between ERS and mitochondrial dysfunction in response to IRI. TCMK-1 cells were transfected with lenti-*Xbp1*, siRNA-*Xbp1*, and corresponding control vectors, respectively. For the cell H/R model, hypoxia was performed in a tri-gas hypoxia incubator for 24 h, and then reoxygenation was performed for 2 h after applying a fresh medium. **A** NLRP3 protein expression correlated with XBP1 protein expression following lenti-*Xbp1* and siRNA-*Xbp1* transfection. Western blot; *P < 0.05, ***P < 0.001 vs. the normal control group (*n* = 3 samples/group). **B** NLRP3 mRNA expression correlated with XBP1 mRNA expression following lenti-*Xbp1* and siRNA-*Xbp1* transfection. Real-time quantitative reverse-transcription PCR; *P < 0.05, **P < 0.01, ***P < 0.001 vs. normal control group (*n* = 3 samples/group). **C** TCMK-1 cells were transfected with siRNA- *Xbp1* or siRNA-control before H/R. NLRP3 protein expression correlated with XBP1 protein expression after H/R, as shown in the western blot and densitometric analysis. *P < 0.05, **P < 0.01, ***P < 0.001 vs. the normal control H/R group. **D** The pGL4.10 firefly luciferase reporter plasmids with the wild-type (WT) NLRP3 promoter were transiently transfected into 293T cells together with the pcDNA3.1(+)-XBP1s plasmid or overexpression (OE) negative control and a Renilla luciferase reporter for normalization. Luciferase activities were measured after 48 h. The results showed that the NLRP3 promoter activity of the XBP1 overexpression group was higher than that of the control group; *P < 0.05. Further details are shown in the “Materials and methods” section. LV-NC lentivirus normal control, si-NC siRNA normal control, H/R hypoxia/reoxygenation.

To further analyze the effect of XBP1 modulation on NLRP3 expression and activity, we transfected TCMK-1 cells with an XBP1-overexpression lentivirus. In these cells, the relative RNA expression of XBP1u was 1.21 ± 0.11 vs. 3.25 ± 0.48, and that of XBP1s was 0.82 ± 0.41 vs. 19.49 ± 5.41. Meanwhile, the protein expression of XBP1u was 0.47 ± 0.06 vs. 0.74 ± 0.06, and that of XBP1s was 0.20 ± 0.04 vs. 0.45 ± 0.06 (all *P* < 0.05). Further, increased XBP1 expression enhanced NLRP3 expression (mRNA, 1.01 ± 0.01 vs. 1.18 ± 0.04; protein, 0.37 ± 0.06 vs. 0.72 ± 0.10; both *P* < 0.05). For TCMK-1 cells transfected with XBP1-targeting siRNA, the values relative RNA expression levels were as follows: for XBP1u, 0.84 ± 0.03 vs. 0.03 ± 0.01, *P* < 0.01; for XBP1s, 1.19 ± 0.09 vs. 0.02 ± 0.01, *P* < 0.001. The relative protein expression values were 0.48 ± 0.05 vs. 0.05 ± 0.01, *P* < 0.001 for XBP1u and 0.17 ± 0.03 vs. 0.03 ± 0.01, *P* < 0.05 for XBP1s. Relative to the empty-vector group, NLRP3 expression was lower in the si-XBP1 group (mRNA: 1.02 ± 0.01 vs. 0.54 ± 0.04, *P* < 0.001; protein: 0.40 ± 0.07 vs. 0.10 ± 0.01, *P* < 0.05) (Fig. 3A, B). Moreover, XBP1s production was inhibited upon treatment with 4μ8c, an IRE1α inhibitor, further reducing NLRP3 expression (Supplementary Fig. S1).

To further verify the effects of XBP1 on NLRP3 under stress conditions, H/R was used to simulate renal IRI in vitro. Our experiments revealed that XBP1 downregulation inhibited NLRP3 expression (XBP1u: 1.24 ± 0.15 vs. 0.34 ± 0.13, *P* < 0.05; XBP1s: 0.27 ± 0.03 vs. 0.03 ± 0.01, *P* < 0.01; NLRP3: 0.97 ± 0.07 vs. 0.22 ± 0.02, *P* < 0.001; Fig. 3C).

We then examined the mechanisms through which XBP1 regulates NLRP3. Based on bioinformatics analysis, we predicted that XBP1 might regulate NLRP3 promoter activity via XBP1s. We conducted a dual-luciferase reporter assay to confirm if XBP1s binds to the NLRP3 promoter. First, we constructed an XBP1s-overexpression plasmid containing the NLRP3 promoter. This, along with a luciferase reporter plasmid, was transfected into 293T cells. Before interaction with the XBP1s transcription factor, NLRP3 promoter activity was 13.52 times higher in the XBP1-overexpression group than in the control group (*P* < 0.05), indicating activation of the NLRP3 gene promoter. After interacting with the XBP1s transcription factor, NLRP3 gene promoter activity was 1.81 times higher than in the control group, indicating that the XBP1s transcription factor enhances NLRP3 gene promoter activity (*P* < 0.05; Fig. 3D). Unfortunately, we obtained negative results for our three predicted binding sites. Therefore, the binding sites require further investigation.

### XBP1 downregulation reduced the production of NLRP3 downstream effectors, mitochondrial damage, and apoptosis in TCMK-1 cells exposed to H/R

**Fig. 4:**
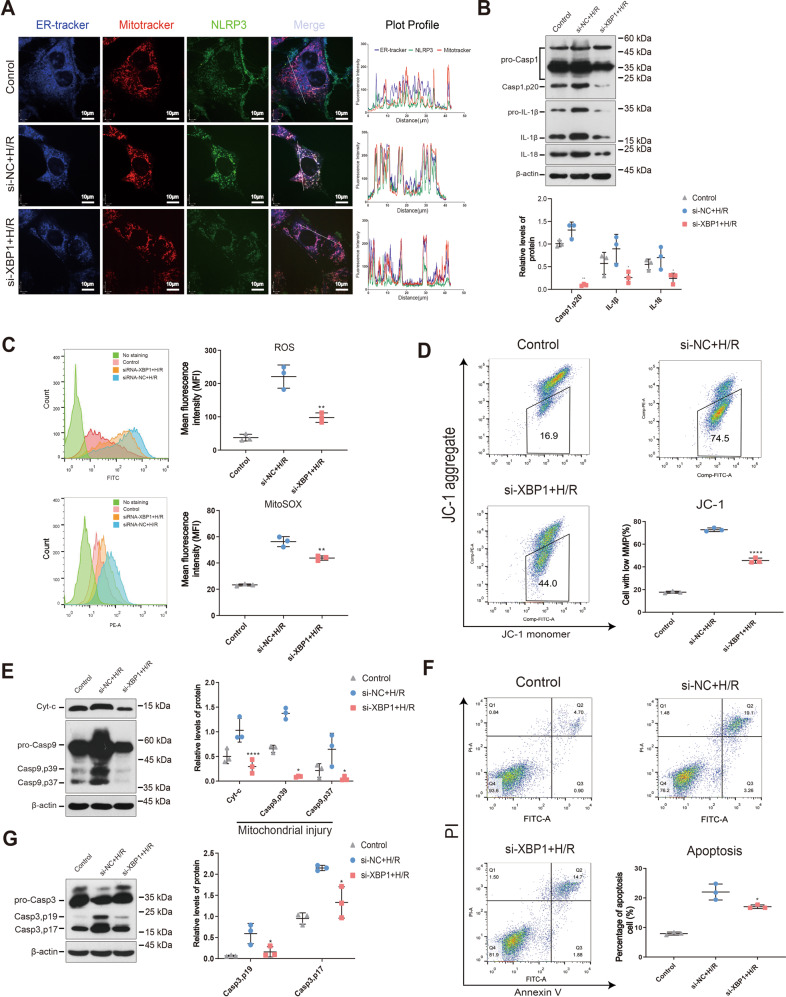
Downregulation of XBP1 ameliorated NLRP3 downstream molecules, mitochondrial damage, and apoptosis in TCMK-1 cells exposed to H/R. TCMK-1 cells were transfected with lenti-*Xbp1*, siRNA-*Xbp1*, and their corresponding control vectors. For the cell H/R model, hypoxia was induced in a tri-gas hypoxia incubator for 24 h, and then reoxygenation was performed for 2 h after adding fresh culture medium. **A** Colocalization of the ER, mitochondria, and NLRP3 after siRNA-*Xbp1* transfection in TCMK-1 cells exposed to H/R as analyzed using confocal microscopy. Plot profile: the histograms (right panels) represent densities along the white bars: blue = ERtracker, red = Mitotracker, green = NLRP3, purple = overlap of ERtracker and Mitotracker, yellow = overlap of NLRP3 and Mitotracker, cyan = overlap of NLRP3 and ERtracker. Scale bar = 10 μm. Original magnification of photograph: ×1000. **B** Silencing of XBP1 led to the inhibition of inflammation (caspase-1, IL-1β, and IL-18) in TCMK-1 cells exposed to H/R. Western blot; *P < 0.05, **P < 0.01 vs. the siRNA-control H/R group (*n* = 3 samples/group). **C** H/R-induced ROS (tROS and mROS) production was significantly attenuated in TCMK-1 cells treated with *Xbp1* siRNA, as shown using flow cytometry. **P < 0.01 vs. the siRNA-control H/R group (*n* = 3 samples/group). **D** Treatment with *Xbp1* siRNA dramatically increased the mitochondrial membrane potential in TCMK-1 cells exposed to H/R, as shown using flow cytometry. *P < 0.05 vs. the siRNA-control H/R group (*n* = 3 samples/group). **E** Total Cyt-c and cleaved caspase-9 expression were reduced by siRNA-*Xbp1* transfection in H/R-exposed TCMK-1 cells. Western blot; *P < 0.05, ****P < 0.0001 vs. the siRNA-control H/R group (*n* = 3 samples/group). **F** The apoptotic proportion of H/R-exposed TCMK-1 cells was decreased by siRNA-*Xbp1* transduction, as revealed using flow cytometry. *P < 0.05 vs. the siRNA-control H/R group (*n* = 3 samples/group). **G** Cleaved caspase-3 (p17 and p19) expression was decreased by siRNA-*Xbp1* transfection in TCMK-1 cells exposed to H/R. Western blot; *P < 0.05 vs. the siRNA-control H/R group (*n* = 3 samples/group). si-NC siRNA normal control, H/R hypoxia/reoxygenation.

After elucidating the possible mechanism whereby XBP1 regulates NLRP3, we examined whether XBP1 downregulation affects the expression of the downstream effector molecules of NLRP3 in vitro. We then verified the changes in NLRP3 localization following XBP1 downregulation using a mitochondrial probe (MitoTracker), ER probe (ER Tracker Blue White DPX dye), and anti-NLRP3 antibodies and observed them via confocal microscopy. Under hypoxia, colocalization with NLRP3 was enhanced in the mitochondria and ER; colocalization at all three sites was reduced after XBP1 downregulation (Fig. 4A). Based on western blot analysis, XBP1 downregulation inhibited inflammation in TCMK-1 cells after 24 h of hypoxia followed by 2 h of reoxygenation, which was not exhibited in the siRNA-treated control (Casp1, p20: 1.31 ± 0.10 vs. 0.10 ± 0.01, *P* < 0.01; IL-1β: 0.99 ± 0.10 vs. 0.26 ± 0.07, *P* < 0.05; IL-18: 0.54 ± 0.06 vs. 0.16 ± 0.03, *P* < 0.05; Fig. 4B).

We then examined the protective effect of XBP1 inhibition on the mitochondria using an in vitro H/R model. To do this, we used a DCFH-DA probe to stain the total reactive oxygen species (tROS) and the mitochondrial superoxide indicator MitoSOX red to stain mitochondrial ROS (mROS). ROS were detected via flow cytometry. MMP was detected via staining with the fluorescent dye JC-1, followed by flow cytometry. The results showed that siRNA-induced XBP1 downregulation reduced ROS production (tROS: 221 ± 20.11 vs. 97.60 ± 8.17, *P* < 0.01; mROS: 56.27 ± 2.21 vs. 43.77 ± 0.98, *P* < 0.01; Fig. 4C), and elevated the MMP (72.77 ± 0.91 vs. 45.53 ± 1.29, *P* < 0.0001; Fig. 4D). These changes did not occur in the si-negative control (NC)-transfected cells. These results indicate that XBP1 inhibition can protect mitochondrial function in an in vitro H/R cell model.

Western blot analysis of Cyt-c and Casp9 expression revealed that the rates of mitochondrial damage were lower in the si-XBP1-treated group than in the control (Casp9, p39: 1.38 ± 0.07 vs. 0.09 ± 0.01, *P* < 0.05; Casp9, p37: 0.65 ± 0.19 vs. 0.05 ± 0.02, *P* < 0.0001; Cyt-c: 1.03 ± 0.14 vs. 0.30 ± 0.08, *P* < 0.05; Fig. 4E).

To detect apoptosis, we conducted Annexin V/PI analysis (22.01 ± 1.56 vs. 17.03 ± 0.36, *P* < 0.05; Fig. 4F) and used western blotting to measure the expression of caspase-3 (Casp3), a key apoptosis marker (Casp3, p19: 0.59 ± 0.14 vs. 0.16 ± 0.07, *P* < 0.05; Casp3, p17: 2.15 ± 0.04 vs. 1.33 ± 0.22, *P* < 0.05; Fig. 4G). The proportion of apoptotic cells was lower in the si-XBP1 group than in the control group.

### Genotyping of Xbp1^+/−^ mice

**Fig. 5:**
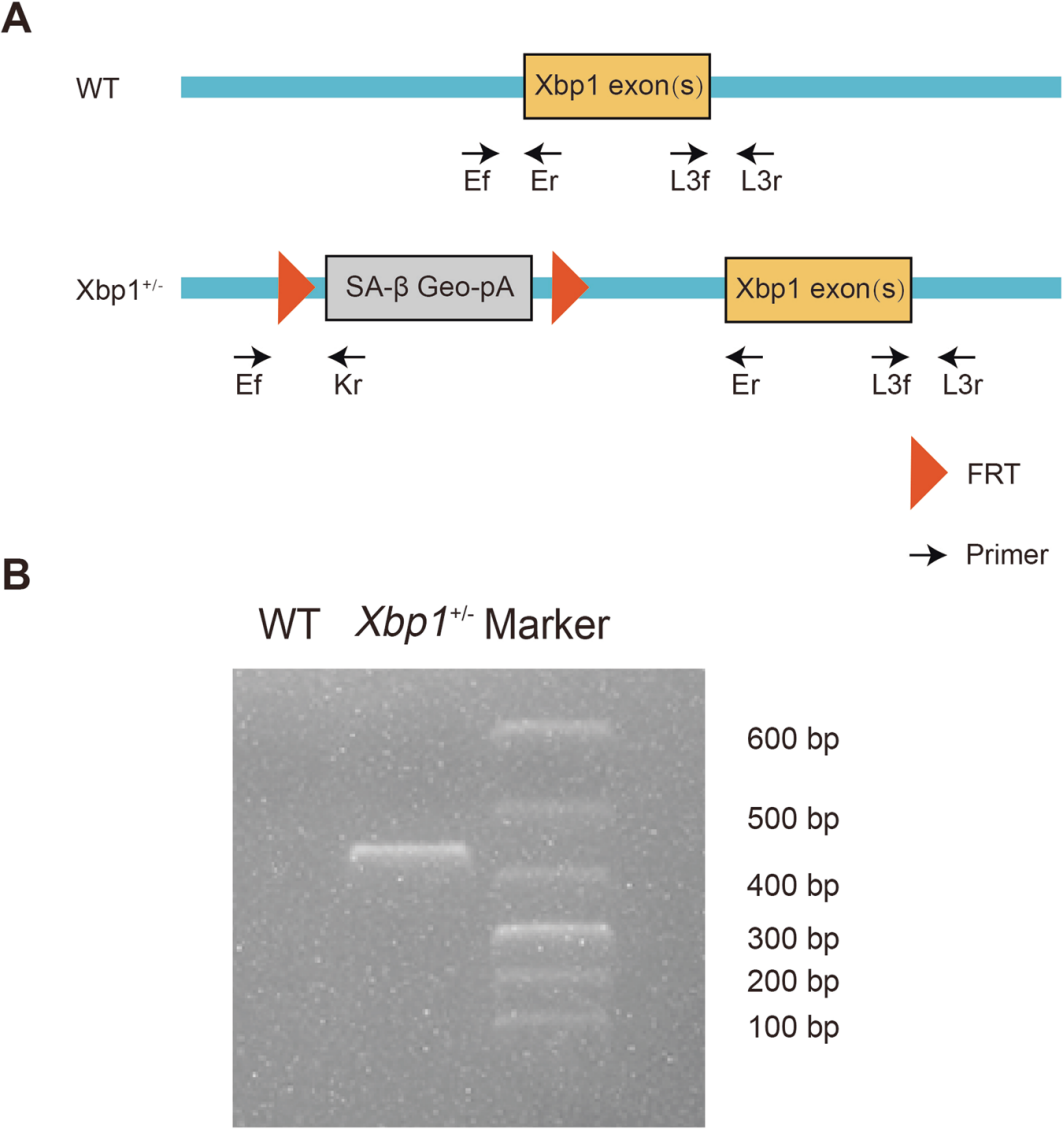
Identification of Xbp1^+/**−**^ mouse genotypes. **A** Schematic representation of the heterozygous Xbp1^+/−^ mouse model. **B** Genotyping of Xbp1^+/−^ mice was authenticated via PCR using Ef and Kr primers.

The heterozygous murine *Xbp1*^+/−^ allele contains a trapping cassette, the splice acceptor-β-geo-polyA (SA-βgeo-pA), which is flanked by the flippase recombination target of the upstream exon. This causes the truncation of endogenous transcripts and constitutive null mutations that reduce XBP1 expression (Fig. 5A). Genotyping of Xbp1^+/−^ mice was confirmed via PCR using Ef and Kr primers (Fig. 5B).

### Downregulation of XBP1-NLRP3 signaling reduced the crosstalk between ERS and mitochondrial damage and protected the kidneys of Xbp1^+/−^mice from IRI

**Fig. 6:**
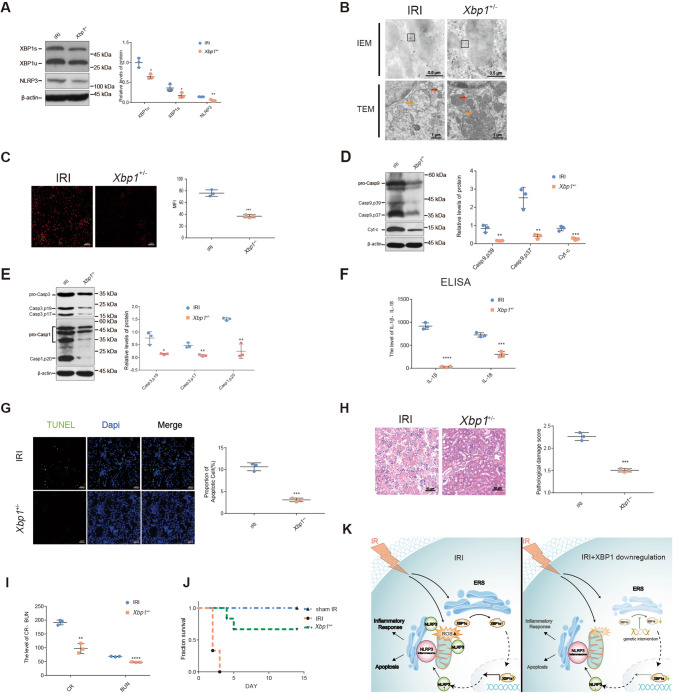
Downregulation of XBP1-NLRP3 ameliorated the crosstalk between ERS and mitochondrial damage and protected the kidneys affected by IRI in Xbp1^+/**−**^mice. To construct the mouse IRI model, we used microvascular clips to block the left renal pedicle of the mouse for 45 min and then cut off the right kidney after opening the bloodstream. **A** Downregulation of XBP1 expression significantly suppressed the protein levels of NLRP3. Western blot; *P < 0.05, **P < 0.01 vs. the IRI group (*n* = 3 samples/group per group). **B** IEM showed that the colocalization of NLRP3 and mitochondria or MAMs induced by IRI was reduced in the kidneys of Xbp1^+/−^ mice. Blank rectangles indicate MAMs, and the small black dots indicate the 10-nm colloidal gold particles Scale bar = 0.5 μm; ×10,000 magnification. IRI-induced ER and mitochondrial damage were significantly alleviated because of XBP1 downregulation, as shown using TEM. The red arrows indicate ER swelling, while the orange arrows indicate the damaged mitochondria. Scale bar = 1 μm; ×5000 magnification. **C** XBP1 downregulation reduced the production of ROS in the kidneys exposed to IRI, as shown via DHE staining. Scale bar = 50 μm; ×200 magnification. **D** Cleaved caspase-9 and total Cyt-c expression were reduced in the kidneys of Xbp1^+/−^ mice after IRI. Western blot; **P < 0.01, ***P < 0.001 vs. the IRI group (*n* = 3 samples/group). **E** XBP1 downregulation alleviated NLRP3-mediated apoptosis (cleaved caspase-3 p17 and p19) and inflammation (caspase-1) in the kidneys exposed to IRI. Western blot; *P < 0.05, **P < 0.01 vs. the IRI group (*n* = 3 samples/group). **F** The ELISA results indicated that the protein level of inflammatory factors IL-1β and IL-18 were decreased in the serum. ***P < 0.001, ****P < 0.0001 vs. the IRI group (*n* = 3 samples/group). **G** Downregulation of XBP1 expression dramatically reduced the ratio of apoptotic cells, as observed using the TUNEL assay. Scale bar = 50 μm; ×200 magnification. ***P < 0.001 vs. the IRI group (*n* = 3 samples/group). **H** Acute renal damage was alleviated in Xbp1^+/−^ mice subjected to IRI, as shown using H&E staining and the pathological damage scores. Scale bar = 50 μm; ×200 magnification. ***P < 0.001 vs. the IRI group (*n* = 3 samples/group). **I** Downregulation of XBP1 expression dramatically reduced the levels of CR and BUN in the serum. **P < 0.01, ****P < 0.0001 vs. the IRI group (*n* = 3 samples/group). **J** Downregulation of XBP1 expression dramatically improved the survival of Xbp1^+/−^ mice. * *P* < 0.05 vs. the IRI group (*n* = 3 samples/group). **K** Schematic illustration of the mechanism involving the XBP1-NLRP3 axis in regulating the ER and mitochondrial crosstalk in renal ischemia/reperfusion. IRI ischemia/reperfusion injury.

To evaluate the importance of the XBP1-NLRP3 axis in the crosstalk between ERS and mitochondrial damage in IR-induced renal inflammation, we studied heterozygous Xbp1^+/−^ mice generated using the knockout-first strategy. XBP1 expression was observed to be somewhat lower in the kidneys of Xbp1^+/−^ mice subjected to ischemia-reperfusion than in the control group, resulting in reduced NLRP3 expression (XBP1u: 1.09 ± 0.039 vs. 0.70 ± 0.04, *P* < 0.01; XBP1s: 0.29 ± 0.01 vs. 0.14 ± 0.01, *P* < 0.001; NLRP3: 0.14 ± 0.01 vs. 0.05 ± 0.01, *P* < 0.01; Fig. 6A). IEM revealed the reduced localization of NLRP3 in the mitochondria or MAMs of Xbp1^+/−^ mice. TEM revealed that the Xbp1^+/−^ mice had more complete mitochondrial structures than the control mice (Fig. 6B). In Xbp1^+/−^ mice, following mitochondrial damage, there was lower production of ROS (76.07 ± 3.19 vs. 36.88 ± 1.60; *P* < 0.001) and Cyt-c (0.84 ± 0.08 vs. 0.26 ± 0.03; *P* < 0.01), and caspase-9 activation was inhibited (Casp9, p39: 0.83 ± 0.12 vs. 0.16 ± 0.01, *P* < 0.01; Casp9, p37: 2.53 ± 0.33 vs. 0.40 ± 0.07, *P* < 0.01) (Fig. 6C, D). Xbp1^+/−^ mice also exhibited a reduced expression of caspase-3 (Casp3, p19: 0.76 ± 0.14 vs. 0.13 ± 0.02, *P* < 0.05; Casp3, p17: 0.47 ± 0.06 vs. 0.07 ± 0.02, *P* < 0.01) and caspase-1 (1.50 ± 0.03 vs. 0.24 ± 0.16, *P* < 0.01). These results reveal that inhibiting NLRP3 expression reduced NLRP3-induced apoptosis and inflammasome activation (Fig. 6E).

We then performed ELISA to examine the expression of IL-1β (915.50 ± 41.19 vs. 31.57 ± 7.33, *P* < 0.0001) and IL-18 (723.90 ± 27.08 vs. 303.60 ± 34.05, *P* < 0.001) in the mouse serum (Fig. 6F). These findings indicate that the NLRP3-inflammasome-induced inflammatory response was greatly attenuated in Xbp1^+/−^ mice.

TUNEL staining revealed renal cell apoptosis was reduced in Xbp1^+/−^ mice (10.65 ± 0.53 vs. 3.10 ± 0.24, *P* < 0.001; Fig. 6G). Compared with the control, XBP1 inhibition in Xbp1^+/−^ mice reduced IR-induced kidney damage and histological scores (0.95 ± 0.21 vs. 3.75 ± 0.35, *P* < 0.01, Fig. 6H), and serum creatinine (191.70 ± 5.04 vs. 97.33 ± 10.17, *P* < 0.01) and blood urea nitrogen levels (68.27 ± 0.64 vs. 47.86 ± 0.64, *P* < 0.0001) (Fig. 6I).

The 14-day survival analysis revealed that Xbp1^+/−^ mice displayed significantly longer survival than wild-type mice that received the same treatment (*P* < 0.001, Fig. 6J).

## Discussion

In this study, we examined how the XBP1-NLRP3 axis modulates ER-mitochondrial crosstalk during renal IRI in vivo and in vitro. To the best of our knowledge, this is the first study to examine the pivotal role of the XBP1-NLRP3 signaling pathway in regulating NLRP3-mediated cell injury in renal IRI. XBP1 increased the expression of the NLRP3 promoter and enhanced its transcriptional activity, which is necessary to induce renal injury. XBP1 knockdown inhibited NLRP3-mediated inflammation, thus regulating the interaction between the mitochondria and MAMs, thereby reducing apoptosis activation and alleviating IRI-induced AKI. These findings highlight the importance of the XBP1-NLRP3 axis in regulating ER-mitochondrial crosstalk in IR-stressed kidneys (Fig. 6K).

Accumulating evidence suggests that the structural and functional crosstalk between the ER and mitochondria contributes to the underlying mechanism of renal IRI [16]. In our mouse model, TEM revealed that the ER from ischemia-reperfusion injured kidneys were dilated, indicating ERS. We observed that the damaged mitochondria were surrounded by abnormal ER; this implies that the mitochondria and ER collaborate in response to AKI during IRI. In addition, we observed that the expression of XBP1s, the activated form of XBP1, was higher than that of other ERS-associated molecules.

Although ER-mitochondrial crosstalk is known to play a crucial role in the kidneys, it remains unclear how the ER affects the mitochondria in IR-mediated renal injury. The NLRP3 inflammasome is induced under mitochondrial oxidative stress [28–30]. Here, consistent with previous studies [28, 31, 32], our results showed that NLRP3 inflammasome expression and activity were elevated in ischemia-reperfused kidneys, which exhibited increased clustering of NLRP3 inflammasomes on the mitochondria and the MAMs. Other studies have reported that NLRP3 is localized primarily in the ER in the resting state and only translocates to the mitochondria or MAMs under stress. In this study, however, IEM analysis revealed that NLRP3 is localized in both the mitochondria and the ER in the resting state. This discrepancy may arise from the fact that the other studies examined immune cells, whereas we used renal tubular epithelial cells. In other words, NLRP3 subcellular localization may vary among cells.

We then examined the specific regulatory mechanisms underlying the interactions between XBP1 and NLRP3. Our lentiviral-mediated transduction experiment revealed that XBP1 overexpression promoted NLRP3 activation in TCMK-1 cells, consistent with our other finding that siRNA-mediated XBP1 downregulation reduced NLRP3 expression. Moreover, the results of our luciferase assay revealed that the expression of the spliced XBP1 isoform enhanced NLRP3 gene promoter activity. More research is essential to further elucidate these mechanisms.

XBP1 knockdown inhibited NLRP3 expression and NLRP3 inflammasome activation, thereby protecting the kidneys from ischemic reperfusion injury to some extent. This is consistent with findings from previous literature showing that the inhibition of XBP1 activity reduced NLRP3 inflammasome assembly and caspase-1 processing in a model of hepatic ischemia-reperfusion, thus reducing IRI-triggered liver injury [33, 34].

Further, Chou et al. [35] found that XBP1s deacetylation or inhibition improved cadmium-induced ERS- and NLRP3-related pyroptosis in human renal tubular epithelial cells. In general, under renal IRI in vivo and H/R in vitro, we found that XBP1 downregulation inhibited NLRP3-related inflammation and apoptosis; this protected the mitochondria by inhibiting the release of mitochondrial pro-apoptotic factors and reducing mitochondrial damage. Although our approach did not directly regulate the NLRP3 inflammasome and ROS, ROS are known to be the primary mediators of NLRP3 inflammasome activation [28, 36]. Reducing the production of mitochondrial ROS during AKI can inhibit NLRP3 inflammasome activation and reduce renal tubular epithelial cell death [37–39]. Moreover, NLRP3 inhibition via XBP1 downregulation can reduce mitochondrial damage in AKI and protect the renal tubules [40]. Apart from its role in inflammasomes, the direct or indirect inhibition of NLRP3 could also help repair tubular epithelial cells and alleviate the pathological processes of renal IRI; this is consistent with the results of our study and of other studies [41, 42]. Our results imply that NLRP3 is a potential target for clinical interventions to reduce IRI.

Based on these findings, we propose a mechanism whereby the XBP1-NLRP3 axis regulates ER-mitochondrial crosstalk in renal ischemia/reperfusion (Fig. 6K). In IRI, IRE1α is induced, which in turn activates the splicing of XBP1 mRNA, promoting XBP1 activation. Subsequently, XBP1s becomes translocated into the nucleus to regulate the NLRP3 promoter, promoting NLRP3 expression. NLRP3 localization in the MAMs or mitochondria contributes to NLRP3-mediated inflammation and activation of apoptosis, and ROS derived from damaged mitochondria activates the NLRP3 inflammasome. Hence, the suppression of XBP1 activity may inhibit NLRP3 and its downstream pathways to prevent IRI-triggered renal injury.

XBP1 has distinctive functions in different tissue types. Arthur et al. reported that XBP1 deficiency induces a pro-inflammatory response and ERS in intestinal epithelial cells, inducing apoptosis [43]. In renal tubule epithelial cells, Silvia et al. found that XBP1s overexpression contributed to renal inflammation and injury, and promoted septic AKI [44], consistent with our findings. Conversely, Claudio et al. found that XBP1 deletion in the nervous system improves the survival of mSOD1G86R transgenic mice by increasing autolysosome production and reducing apoptosis [45]. Further research is required to examine the roles of XBP1 in different tissue types and microenvironments.

These findings have important potential clinical and translational applications. In the short term, the most promising translational strategy may be to examine whether the XBP1-NLRP3 signaling pathway drives AKI in patients and whether it is a druggable target for clinical treatment. In the long term, further research is required to develop a comprehensive understanding of XBP1 as a predictive biomarker for early IR-related AKI diagnosis and as a blockade target to improve the overall survival of patients with AKI.

## Materials and methods

### Ethics approval

The study was approved by the Animal Care and Use Committee of Tongji Medical College and Nanfang Hospital, Southern Medical University. The experiments were performed in accordance with the relevant guidelines and regulations of this committee.

### Mice

Beijing Weitong Lihua Experimental Animal Technology Co., Ltd. (China) provided the C57BL/6 mice used in this study. Heterozygous Xbp1^+/−^ C57BL/6 mice (C57BL/6NTac-XBP1 < tmla<EUCOMM > Wtsi > /1cs0r1) were generated by the European Mouse Mutant Cell Repository (Phil Avner, Paris, France). All animals were raised in specific pathogen-free rooms (at 22 °C, 55% humidity, and under a 12/12 h light/dark cycle) at Tongji Hospital. The primers used to identify the mouse genotypes are listed in Supplementary Table S1.

### Establishment of a renal IRI mouse model

Briefly, 8–10 week-old male mice weighing 22 ± 2 g were anesthetized via intraperitoneal injection of pentobarbital (50 mg/kg) and placed on a heating pad at 32 °C. A midline abdominal incision was made to expose the left kidney, and a noninvasive arterial clamp was used to clamp the left renal pedicle to cause renal ischemia. Afterward, the mice were placed in a constant temperature incubator at 32 °C. The vascular clamp was released after 45 min to simulate reperfusion. Meanwhile, the right kidney was removed, and the incision was sutured layer by layer. Blood and kidney samples were collected after 24 h. The control group was fed normally to observe their survival rate.

### Histopathological analysis

For each slide, 10 fields of vision were randomly selected under a light microscope (at ×200 magnification) to observe the shape, nuclear staining state, and lumen dilation of the renal tubular epithelial cells in each visual field. The results were graded from 0 to 3 with the following criteria: 0, no injury; 1, mild injury, presence of round epithelial cells and tubule lumen; 2, severe injury, epithelial cells flattened, nuclear staining no longer detectable, lumenal dilation, and lumenal hyperemia; 3, renal tubule destruction, epithelial cells flattened, nuclear staining no longer detectable, and lumenal hyperemia.

### ROS detection

Renal tissue was prepared as frozen sections. An ROS staining working solution was added to the marked area, and the sections were incubated at 37 °C for 30 min, protected from light. The sections were then washed thrice with PBS on a shaker. The sections were then incubated with a DAPI solution at room temperature for 10 min to stain the nuclei. After washing, samples were observed under a fluorescence microscope (Nikon Eclipse C1, Tokyo, Japan) and photographed.

### Transmission electron microscopy

Renal tissues were cut into small pieces (1 mm × 1 mm × 2 mm), fixed for at least 4 h in 2.5% glutaraldehyde at 4 °C, rinsed, and treated with 1% osmium tetroxide for 2 h. After washing, renal tissue was dehydrated using a graded ethanol series. After penetration, embedding, and polymerization, ultrathin sections (60–80 nm) were processed using a Leica EM UC7 ultramicrotome (Leica, Wetzlar, Germany) followed by lead and uranium staining for electron microscopy (Tecnai G2 F20 TWIN; FEI Company, Hillsboro, OR).

### Treatment with 4μ8C

The IRE1α inhibitor 4μ8C was purchased from MedChemExpress (CAS-number 14003-96-4; Monmouth Junction, NJ, USA), dissolved in DMSO, and added to the culture medium for 24 h (final concentration, 30 μM).

### Immunoelectron microscopy

Fresh mouse renal tissue samples (1 mm^3^) were fixed using an IEM fixative (G124-10ML; Servicebio, Wuhan, China) overnight at 4 °C. After rinsing with pre-cooled 0.1 M phosphate buffer (PB) (3 times × 10 min each time), the tissue samples were dehydrated in a graded ethanol series. After penetration, embedding, and polymerization, ultrathin sections (70–80 nm) were processed using a Leica EM UC7 ultramicrotome and deposited on a nickel grid with a formvar film for immunolabeling. The grids prepared for immunolabeling were washed with Tris-buffered saline (TBS) droplets (room temperature; 3 times × 10 min each time) and blocked with 1% bovine serum albumin (BSA) in TBS (room temperature, 30 min). For NLRP3 single-labeling, samples were incubated with 1:50 anti-NLRP3 rabbit antibody (ab214185; Abcam, Cambridge, UK) in diluent overnight at 4 °C. The grids were returned to room temperature the next day before rinsing with TBS droplets (room temperature, for 3 × 5 min). Next, the samples were incubated with secondary antibodies conjugated with gold particles (10 nm; G7402; Sigma Aldrich, St Louis, MO), diluted in diluent (room temperature, for 20 min), incubated for 1 h at 37 °C, and finally incubated for another 30 min at room temperature. After immunolabeling, the grids were washed with TBS droplets (room temperature; 5 times × 5 min each time) and rinsed with ultrapure water (room temperature; 5 times × 5 min each time). After rinsing, the samples were stained with a saturated alcohol solution of 2% uranium acetate in the dark for 8 min, followed by three washes in 70% ethanol and three washes in ultrapure water. After drying, the samples were observed and photographed using an HT7800 transmission electron microscope (HITACHI, Japan).

### TUNEL assay

Cell death was analyzed via terminal deoxynucleotidyl transferase (TdT)-mediated dUTP digoxigenin nick-end labeling (TUNEL) in frozen kidney sections using an In Situ Cell Death Detection Kit (11684817910; Roche, Basel, Switzerland).

### ELISA

Serum IL-1β and IL-18 levels were measured using commercially available ELISA kits according to their respective manufacturer’s instructions (Mouse IL-18 ELISA Kit, ab216165, Abcam; Mouse IL-1β ELISA kit, DKW12-2012-096, Dakewe Biotech, China).

### Cell culture and hypoxia-reoxygenation (H/R) model

The TCMK-1 cell line (CCL-139; American Type Culture Collection) was cultured in RPMI 1640 medium (Hyclone; GE Healthcare, Logan, UT, USA) supplemented with 10% fetal bovine serum (Gibco; Thermo Fisher Scientific, Waltham, MA, USA) in a 5% CO_2_ atmosphere. For the hypoxia cell model, TCMK-1 cells were cultured in a pre-hypoxia serum-free medium and incubated in a tri-gas incubator (1% O_2_, 5% CO_2,_ and 94% N_2_) at 37 °C for 24 h (Don Whitely Scientific, Bingley, UK). During the reoxygenation process, the spent medium was replaced with fresh complete medium, and the cells were cultured under normoxic (21% O_2_) conditions for 2 h.

### Immunofluorescence staining and confocal microscopy

The cells were seeded into 15 mm confocal dishes, and immunofluorescence staining was performed after the intervention. MitoTracker Red CMXRos (Life Technologies, Carlsbad, CA, USA), diluted in serum-free medium to 100 nM, was added to the cells, which were then incubated in the dark at 37 °C for 30 min. ER Tracker Blue White DPX dye (Life Technologies, Carlsbad, CA, USA), diluted in serum-free medium to a final concentration of 200 nM, was then added to the cells, which were incubated in the dark at 37 °C for 30 min. Next, the cells were fixed with 4% paraformaldehyde for 15 min and permeabilized using 0.3% after washing three times with PBS. Triton for 15 min, and sealed with 5% BSA for 30 min. Primary antibodies (1:100) were added. The cells were incubated in the dark at 4 °C overnight. DyLight 488 goat anti-rabbit IgG (1:200; A23220; Abbkine, Wuhan, China) was then added, and the cells were incubated in the dark at 37 °C for 1 h. Images were captured using a TiE confocal microscope (Nikon, Tokyo, Japan). A plot profile was generated using ImageJ software to assess the degree of colocalization.

### XBP1 siRNA knockdown and overexpression

Lentivirus-overexpressing XBP1 particles were purchased from Hanbio Biotechnology Co., Ltd. (Shanghai, China). TCMK-1 cells were seeded at a density of 2 × 10^5^ cells/well in six-well plates 12 h before transfection. siRNA transfection was performed using Lipofectamine 2000 (Invitrogen; Thermo Fisher Scientific, Inc., Waltham, MA, USA), and treatments were performed 24 h after transfection. The lentiviral solution (1 × 10^9^ TU/mL) was added to the culture medium (40 μL/well, 4 × 10^7^ TU) at a 50% multiplicity of infection. After 24 h, the spent medium was replaced and the cells were cultured for another 24 h. Stable XBP1-overexpressing strains were screened using a culture medium containing 3 μg/mL puromycin. The siRNA sequences used in this study can be found in Supplementary Table S2.

### Western blotting

Protein quantification was performed using ImageJ software. The antibodies used are listed in Supplementary Table S3.

### Flow cytometry

TCMK-1 cells were cultured in six-well plates and harvested using trypsin without EDTA before and after staining. Cellular ROS, mitochondrial ROS, apoptosis, and MMP were determined using a Reactive Oxygen Species Assay kit (Beyotime Institute of Biotechnology, Shanghai, China), MitoSOX kit (Life Technologies, Carlsbad, CA, USA), Annexin V-PI staining kit, and JC-1 detection kit ((Beyotime Institute of Biotechnology, Shanghai, China), respectively. The cells were then stained according to the respective manufacturer’s instructions and analyzed via flow cytometry. Ten thousand events were recorded using the FACSCalibur system (BD Biosciences, CA, USA), and the resulting data were analyzed using FlowJo software.

### Real-time quantitative reverse-transcription PCR

Total cell RNA was extracted, and PCR was conducted using the appropriate reaction systems. The total RNA obtained was reverse-transcribed into cDNA (15 min at 37 °C, 5 s at 85 °C, indefinite hold at 4 °C). The obtained cDNA stock solution was diluted to 1/8 of its original concentration and amplified according to the instructions of the real-time quantitative PCR MIX kit. The reaction solution was subjected to the following conditions: pre-denaturation at 94 °C for 30 s, then 40 cycles at 94 °C for 5 s and 60 °C for 30 s. The relative mRNA expression levels were then analyzed using β-actin as the internal reference gene. The primer sequences used in this experiment can be found in Supplementary Table S4.

### Dual-luciferase assay

Healthy 293T cells were seeded in 24-well culture plates one day before plasmid transfection. All cell transfection experiments were performed using X-tremeGENE HP DNA Transfection Reagent (Roche, Basel, Switzerland). The expression of fluorescently labeled genes was observed under a fluorescence microscope 48 h after transfection. Luciferase expression was then evaluated using a Dual-Luciferase Reporter Assay System (Promega, Madison, WI). Information about the plasmids and treatment groups can be found in Supplementary Table S5.

### Statistical analysis

All analyses were performed using GraphPad Prism 8 software (La Jolla, CA). Normally distributed data were expressed as the mean ± standard error of the mean. Survival data were compared using the log-rank test. Comparisons among groups were performed using a *t*-test (between two groups) or ANOVA (among more than two groups).

## Supplementary information

Supplementary Figure S1

Supplementary Table S1

Supplementary Table S2

Supplementary Table S3

Supplementary Table S4

Supplementary Table S5

Full western blot images

## Data Availability

Full images of western blotting had been included in Supplementary Materials. Other data are available from the corresponding authors for reasonable grounds.
