# Supplementary material for: XBP1 modulates endoplasmic reticulum and mitochondria crosstalk via regulating NLRP3 in renal ischemia/reperfusion injury
Source: Cell Death Discov. 2023 Feb 17;9:69. doi: 10.1038/s41420-023-01360-x (PMC9938143; doi:10.1038/s41420-023-01360-x)
Supplement: Supplementary file 1 — Supplementary Figure S1 [file 41420_2023_1360_MOESM1_ESM.docx]

**Supplementary Figure S1**


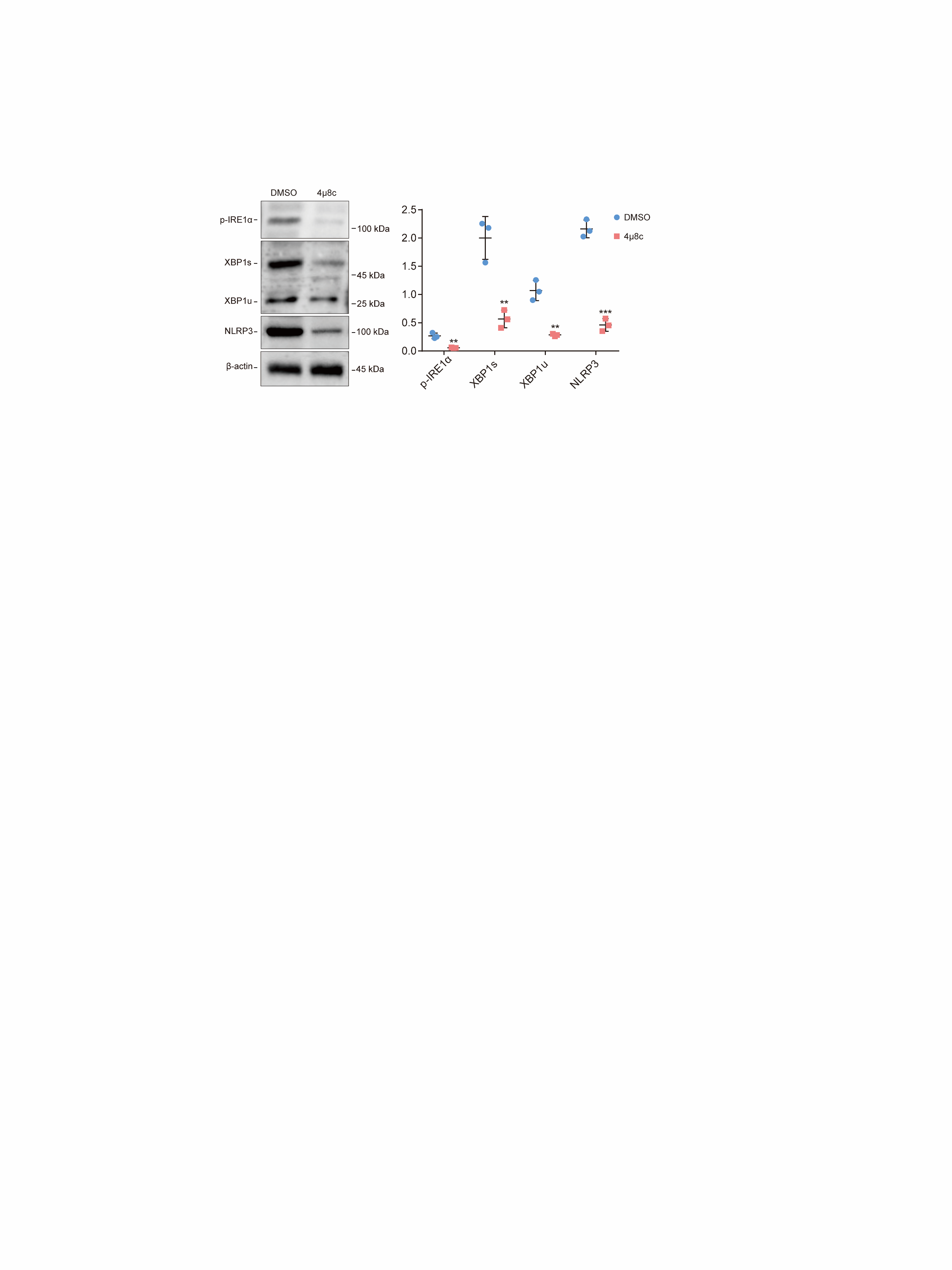
After treating TCMK-1 cells with IRE1α inhibitor 4µ8C (30 μmol/L) for 24 hours, the phosphorylation level of IRE1α and the expression of XBP1s and NLRP3 were significantly inhibited.
