# Supplementary material for: XBP1 modulates endoplasmic reticulum and mitochondria crosstalk via regulating NLRP3 in renal ischemia/reperfusion injury
Source: Cell Death Discov. 2023 Feb 17;9:69. doi: 10.1038/s41420-023-01360-x (PMC9938143; doi:10.1038/s41420-023-01360-x)
Supplement: Supplementary file 2 — Supplementary Table S1 [file 41420_2023_1360_MOESM2_ESM.docx]

**Supplementary Table S1: Primer sequences used for PCR genotyping assay.**

| Primer Position | Sequence |
| --- | --- |
| Er | GGCTTAGCAAGTAAACACGCTTAAAGCTC |
| Ef | GACGGAATTGGACCCAGAAAGTAGC |
| Kr | CTCCTACATAGTTGGCAGTGTTTGGG |
| L3r | CACTGACAGCAGAAAGGATACAGAGG |
| L3f | CCCTCGGAGATTAGCCAGGTTG |
