# Supplementary material for: XBP1 modulates endoplasmic reticulum and mitochondria crosstalk via regulating NLRP3 in renal ischemia/reperfusion injury
Source: Cell Death Discov. 2023 Feb 17;9:69. doi: 10.1038/s41420-023-01360-x (PMC9938143; doi:10.1038/s41420-023-01360-x)
Supplement: Supplementary file 3 — Supplementary Table S2 [file 41420_2023_1360_MOESM3_ESM.docx]

**Supplementary Table S2:** Sequences of siRNA

| Gene | Sequence |
| --- | --- |
| *Xbp1* | 5'-CAGCGCAGACTGCTCGAGATAGAAA-3' |
