# Supplementary material for: XBP1 modulates endoplasmic reticulum and mitochondria crosstalk via regulating NLRP3 in renal ischemia/reperfusion injury
Source: Cell Death Discov. 2023 Feb 17;9:69. doi: 10.1038/s41420-023-01360-x (PMC9938143; doi:10.1038/s41420-023-01360-x)
Supplement: Supplementary file 4 — Supplementary Table S3 [file 41420_2023_1360_MOESM4_ESM.docx]

**Supplementary Table S3:** Details of antibodies used.

| Antibodies | Source (catalog number) | Dilution |
| --- | --- | --- |
| Primary antibody |  |  |
| anti-XBP1 antibody | Abcam (ab37152) | 1:1000 for Western blot |
| anti-NLRP3 antibody | Abcam (ab214185) | 1:500 for Western blot |
| anti-NLRP3 antibody | Abcam (ab214185) | 1:100 for Immunolabeling |
| anti-NLRP3 antibody | Invitrogen (MA5-32255) | 1:100 for Immunofluorescence |
| anti-Caspase-1 antibody | Cell Signaling Technology (2225) | 1:1000 for Western blot |
| anti-Caspase-3 antibody | Cell Signaling Technology (9665) | 1:1000 for Western blot |
| anti-Caspase-9 antibody | Cell Signaling Technology (9504) | 1:1000 for Western blot |
| anti-Caspase-2 antibody | Abcam (ab179520) | 1:1000 for Western blot |
| anti-IL-1β antibody | Cell Signaling Technology (31202) | 1:1000 for Western blot |
| anti-IL-18 antibody | Abcam (ab207323) | 1:1000 for Western blot |
| anti-Cyt-c antibody | Proteintech (10993-1-AP) | 1:1000 for Western blot |
| anti-IRE1α antibody | Cell Signaling Technology (3294) | 1:1000 for Western blot |
| anti-PERK antibody | Cell Signaling Technology (3192) | 1:1000 for Western blot |
| anti-p-PERK antibody | Biss (bs-3330R) | 1:500 for Western blot |
| anti-NRF2 antibody | Abcam (ab137550) | 1:500 for Western blot |
| anti-p-ASK1 antibody | Biss (bs-3007R) | 1:500 for Western blot |
| anti-ATF4 antibody | Proteintech (10835-1-AP) | 1:1000 for Western blot |
| anti-ATF6 antibody | Abcam (ab203119) | 1:1000 for Western blot |
| anti-Chop antibody | Cell Signaling Technology (5554S) | 1:500 for Western blot |
| anti-Bip antibody | Cell Signaling Technology (3177S) | 1:1000 for Western blot |
| anti-β-actin antibody | Abclonal (AC026) | 1:10000 for Western blot |
| anti-phospho-IRE1α-S724 | Novus (NB100-2323) | 1:1000 for Western blot |
| Secondary antibody |  |  |
| Goat anti-rabbit HRP | Proteintech (SA00001-2) | 1:2000 for Western blot |
| Goat anti-mouse HRP | Proteintech (SA00001-1) | 1:2000 for Western blot |
| 10 nm labeled anti-rabbit antibody | Sigma (G7402) | 1:50 for immunolabeling |
