# Supplementary material for: XBP1 modulates endoplasmic reticulum and mitochondria crosstalk via regulating NLRP3 in renal ischemia/reperfusion injury
Source: Cell Death Discov. 2023 Feb 17;9:69. doi: 10.1038/s41420-023-01360-x (PMC9938143; doi:10.1038/s41420-023-01360-x)
Supplement: Supplementary file 5 — Supplementary Table S4 [file 41420_2023_1360_MOESM5_ESM.docx]

**Supplementary Table S4:** Primer sequences used for qPCR analysis.

| Gene | Forward | | Reverse |
| --- | --- | --- | --- |
| *Xbp1*s | 5'-AAGAACACGCTTGGGAATGG-3' | 5'-CTGCACCTGCTGCGGAC-3' | |
| *Xbp1*u | 5'-GTCCATGGGAAGATGTTCTGG-3' | 5'-CAGCACTCAGACTATGTGCA-3' | |
| *Nlrp3* | 5'-ATGGCTGTGTGGATCTTTGC-3 | 5'-CACGTGTCATTCCACTCTGG-3' | |
| *β-actin* | 5'-AGGCCAACCGTGAAAGATG-3' | 5'-TGGCGTGAGGGAGAGCATAG-3' | |
