# Supplementary material for: XBP1 modulates endoplasmic reticulum and mitochondria crosstalk via regulating NLRP3 in renal ischemia/reperfusion injury
Source: Cell Death Discov. 2023 Feb 17;9:69. doi: 10.1038/s41420-023-01360-x (PMC9938143; doi:10.1038/s41420-023-01360-x)
Supplement: Supplementary file 6 — Supplementary Table S5 [file 41420_2023_1360_MOESM6_ESM.docx]

**Supplementary Table S5: Plasmid information and grouping in dual-luciferase assay**

Plasmid

| Code | Name |
| --- | --- |
| BVJ05 | pGL4.10 |
| BW1548 | pGL4.10-Nlrp3-p-wt |
| BVA03 | pcDNA3.1(+) |
| BW1553 | pcDNA3.1(+)-Xbp1s |
| BVJ10 | pRL-TK |

Grouping Information

| Group | Plasmid 1 | Plasmid 2 | Internal reference |
| --- | --- | --- | --- |
| Group 1 | BVJ05 | BVA03 | BVJ10 |
| Group 2 | BVJ05 | BW1553 | BVJ10 |
| Group 3 | BW1548 | BVA03 | BVJ10 |
| Group 4 | BW1548 | BW1553 | BVJ10 |
| Quantity | 1 µg/well | 1 µg/well | 100 ng/well |
