# Supplementary material for: XBP1 modulates endoplasmic reticulum and mitochondria crosstalk via regulating NLRP3 in renal ischemia/reperfusion injury
Source: Cell Death Discov. 2023 Feb 17;9:69. doi: 10.1038/s41420-023-01360-x (PMC9938143; doi:10.1038/s41420-023-01360-x)

Fig 1

NC IRI

BIP


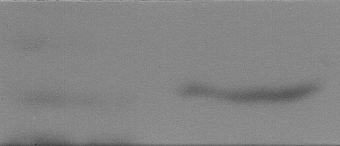


PERK


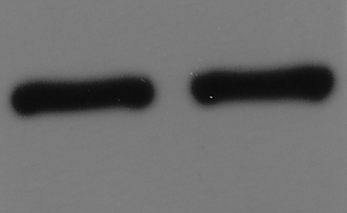


p-PERK


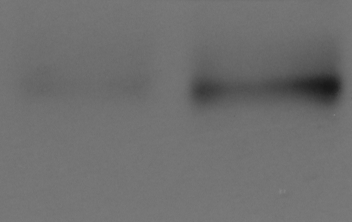


CHOP


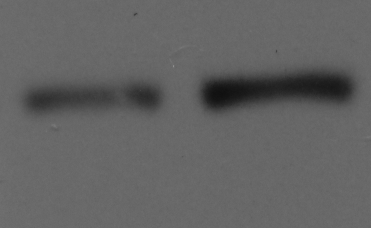


ATF4


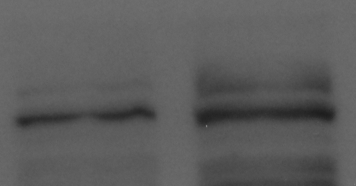


NRF2


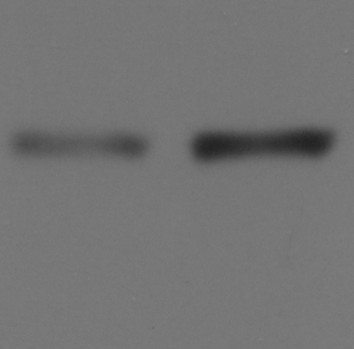


IRE1α


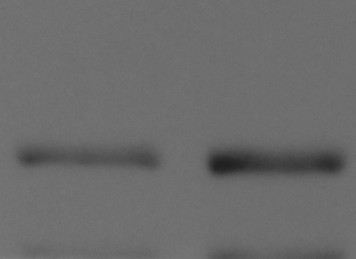


XBP1


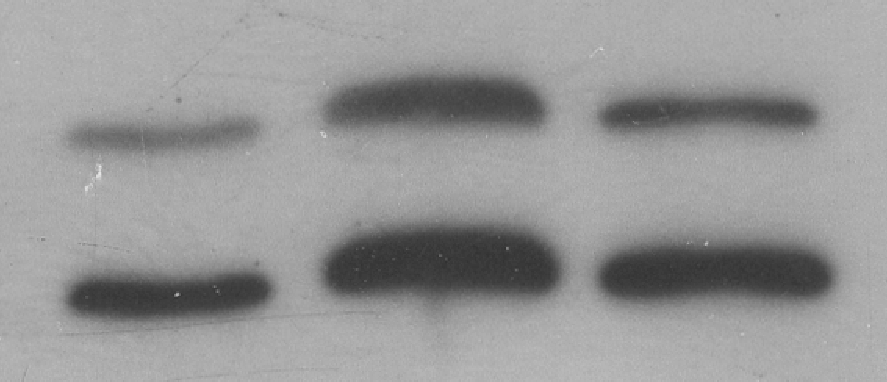


p-ASK1


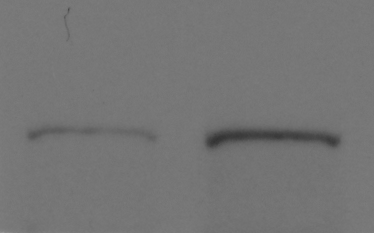


ATF6


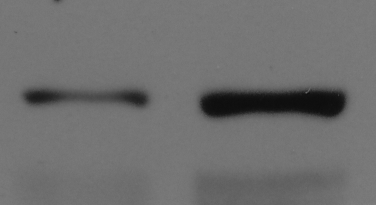


Actin


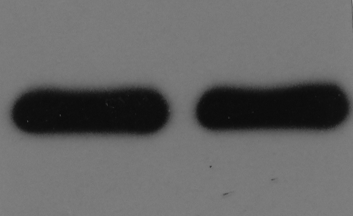


Fig 2

Caspase-9

NC IRI *Xbp1^+/-^*


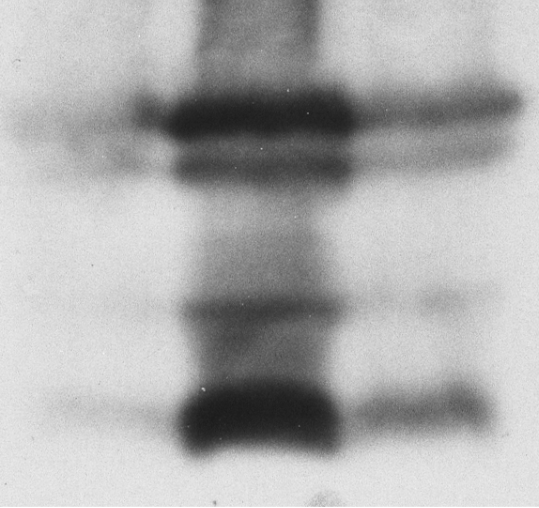


Cyt-c


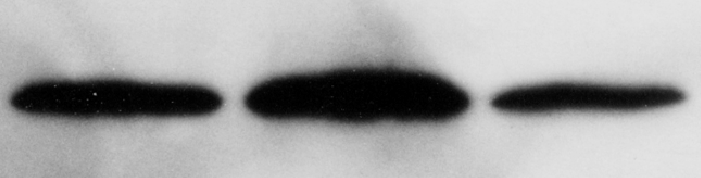


NLRP3


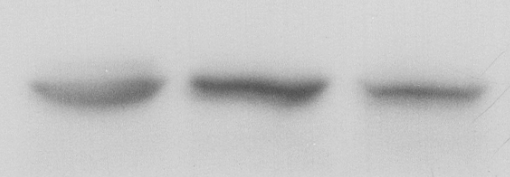


Caspase-1


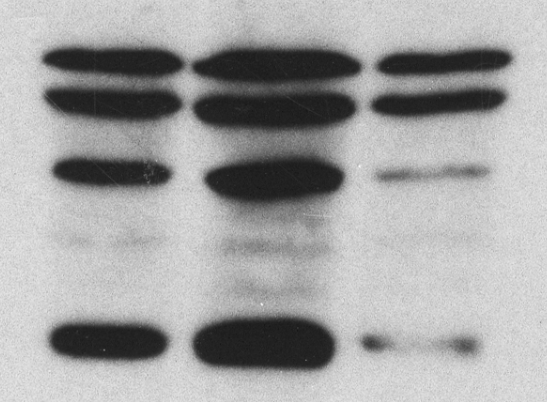


Fig 3

XBP1

LV-NC LV-XBP1 si-NC si-XBP1


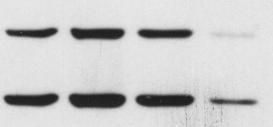


LV-NC LV-XBP1 si-NC si-XBP1

NLRP3


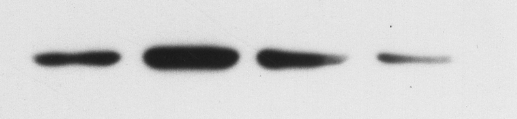


XBP1

NC si-NC+H/R si-XBP1+H/R


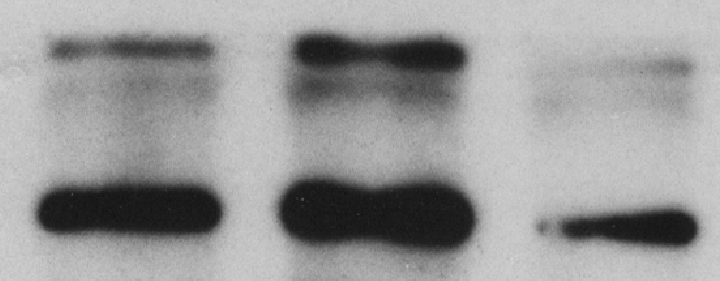


NLRP3

NC si-NC+H/R si-XBP1+H/R


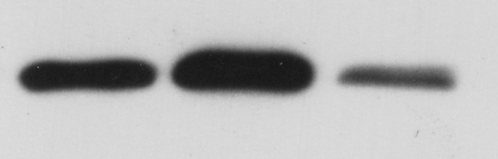


Fig 4

Caspase-1

NC si-NC+H/R si-XBP1+H/R


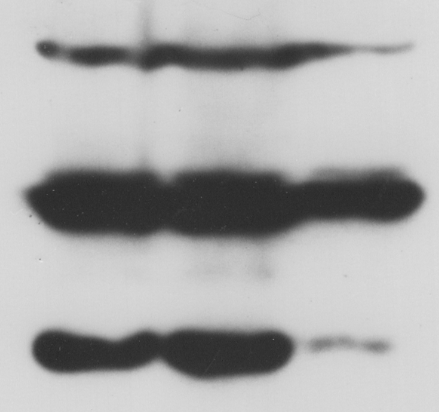


IL-1β


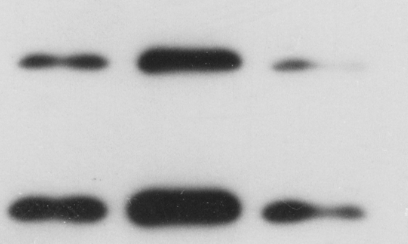


IL-18


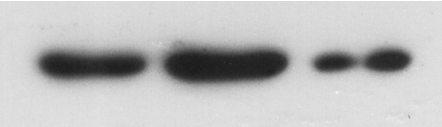


Caspase-2


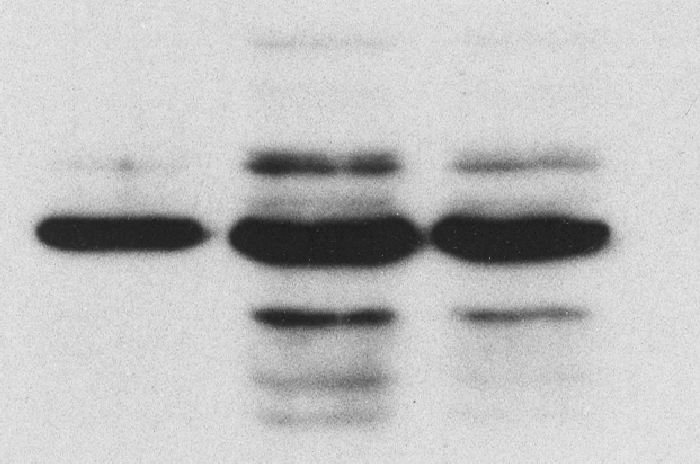


Cyt-c


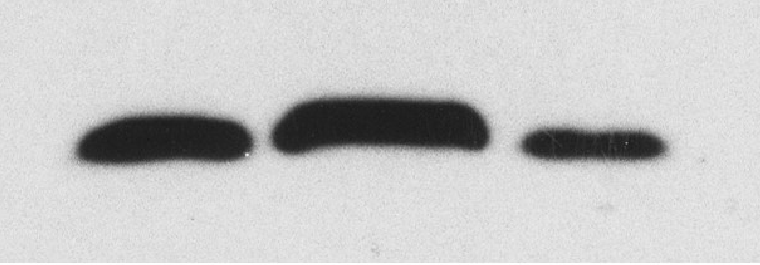


Caspase-9


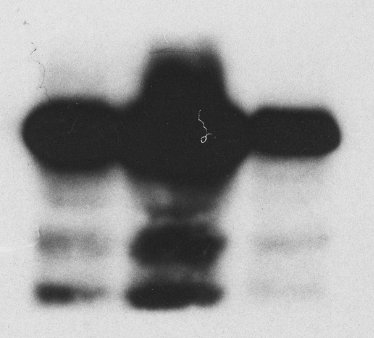


Caspase-3


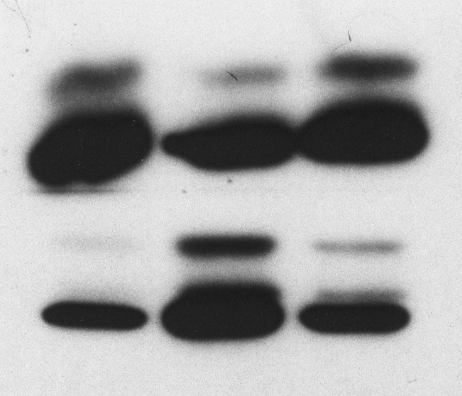


Fig 6

XBP1

NC IRI *Xbp1^+/-^*


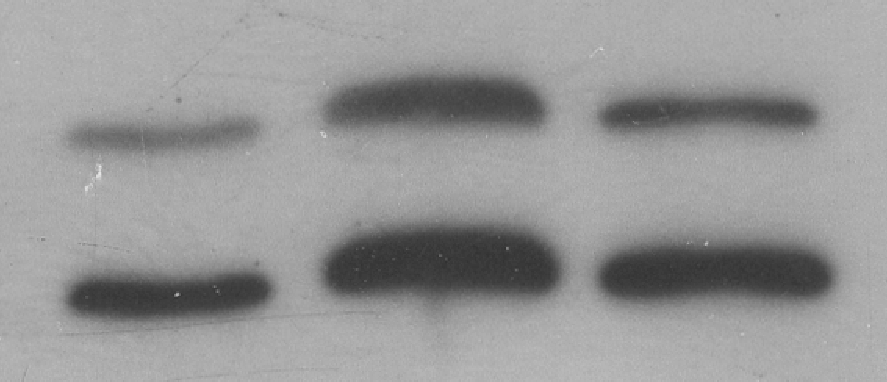


NLRP3


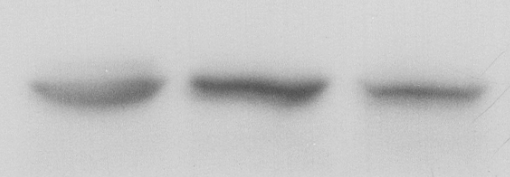


Caspase-9


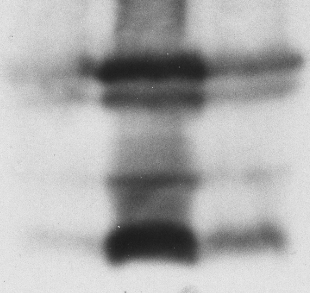


Cyt-c


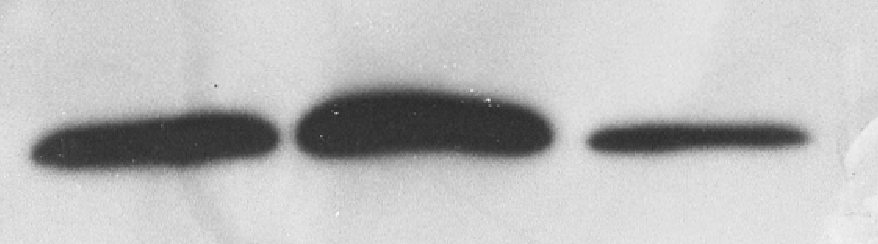


Caspase-2


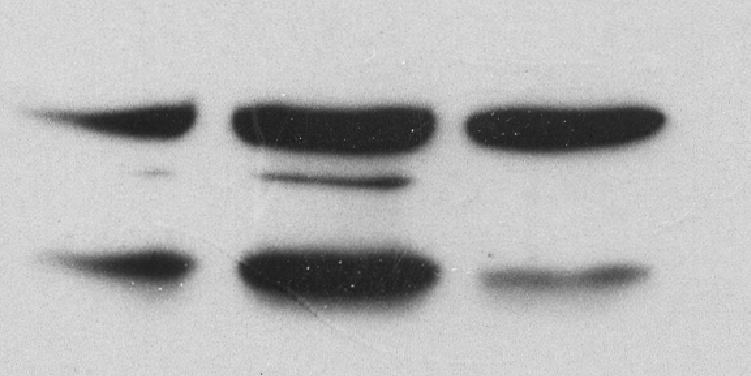


Caspase-3


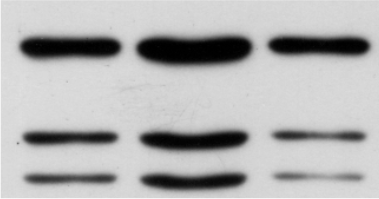


Caspase-1


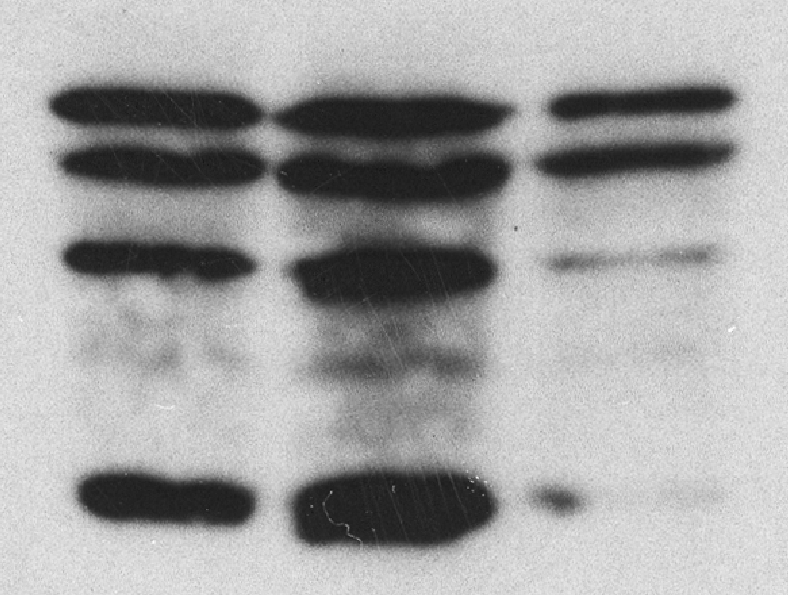

Supplement: Supplementary file 7 — Full western blot images [file 41420_2023_1360_MOESM7_ESM.docx]
